# Aucubin slows the development of osteoporosis by inhibiting osteoclast differentiation via the nuclear factor erythroid 2-related factor 2-mediated antioxidation pathway

**DOI:** 10.1080/13880209.2021.1996614

**Published:** 2021-11-10

**Authors:** Yongfeng Zhang, Xin Liu, Yangyang Li, Minkai Song, Yutong Li, Anhui Yang, Yaqin Zhang, Di Wang, Min Hu

**Affiliations:** a Department of Orthodontics, School and Hospital of Stomatology, Jilin University, Changchun, China; b School of Life Sciences, Jilin University, Changchun, China

**Keywords:** Natural active monomer, metabolic bone disease, reactive oxygen species

## Abstract

**Context:**

Osteoporosis (OP) is a metabolic disease. We have previously demonstrated that aucubin (AU) has anti-OP effects that are due to its promotion of the formation of osteoblasts.

**Objectives:**

To investigate the mechanisms of anti-OP effects of AU.

**Materials and methods:**

C57BL/6 mice were randomly divided into control group, 30 mg/kg Dex-induced OP group (OP model group, 15 μg/kg oestradiol-treated positive control group, 5 or 45 mg/kg AU-treated group), and 45 mg/kg AU-alone-treated group. The administration lasted for 7 weeks. Subsequently, 1, 2.5 and 5 µM AU were incubated with 50 ng/mL RANKL-induced RAW264.7 cells for 7 days to observe osteoclast differentiation. The effect of AU was evaluated by analysing tissue lesions, biochemical factor and protein expression.

**Results:**

The LD_50_ of AU was greater than 45 mg/kg. AU increased the number of trabeculae and reduced the loss of chondrocytes in OP mice. Compared to OP mice, AU-treated mice exhibited decreased serum concentrations of TRAP5b (19.6% to 28.4%), IL-1 (12.2% to 12.6%), IL-6 (12.1%) and ROS (5.9% to 10.7%) and increased serum concentrations of SOD (14.6% to 19.4%) and CAT (17.2% to 27.4%). AU treatment of RANKL-exposed RAW264.7 cells decreased the numbers of multi-nuclear TRAP-positive cells, reversed the over-expression of TRAP5, NFATc1 and CTSK. Furthermore, AU increased the expression of nuclear factor erythroid 2-related factor 2 (Nrf2) and its downstream proteins in RANKL-exposed RAW264.7 cells.

**Conclusions:**

AU slows the development of OP via Nrf2-mediated antioxidant pathways, indicating the potential use of AU in OP therapy and other types of OP research.

## Introduction

Osteoporosis (OP) is a systemic bone metabolic disease that is characterised by a decrease in bone density and the destruction of bone tissue microstructure, which increases the risk of fractures (Zhuang et al. 2020). According to a survey by the World Health Organisation (WHO), with the ageing of the global population, approximately 62% of men and 72% of women aged over 50 years are predicted to have various stages of OP by 2022 (Zamani et al. 2018).

OP is caused by an imbalance between bone formation and bone resorption, which in women is associated with post-menopausal oestrogen deficiency (Wang et al. 2017). Bone remodelling is essential to maintain the stability of bone structure and function (Vasikaran 2018), and osteoblasts, which produce new bone tissue, and osteoclasts, which break down bone tissue, are responsible for the dynamic maintenance of bone homeostasis. Osteoblasts are mononucleated cells that originate from mesenchymal stem cells, whereas osteoclasts are multinucleated cells that originate from haematopoietic stem cells. Osteoblasts and osteoclasts thus maintain a balance between the absorption and formation of bones tissue, based on inflammation levels, oxidation states and hormone changes within the human body (Scheffler et al. 2020).

Oxidative stress is characterised by the overproduction of reactive oxygen species (ROS) due to an imbalance between oxidants and antioxidants (Cai et al. 2019). ROS play a key role in the onset and progression of OP (Zhou et al. 2016), as they inhibit osteoblast differentiation and stimulate osteoclast differentiation, which ultimately leads to an over-resorption of bone tissue (Fraser et al. 1996; Lee et al. 2005).

The drugs that are currently used for the prevention and/or treatment of OP include bisphosphonates, raloxifene and bazedoxifene, but these have various adverse effects, such as gastrointestinal dysfunction (Compston et al. 2017). To develop anti-osteoporotic drugs with strong pharmacological effects and low toxicity that could efficiently reduce the risk of fractures in patients, researchers have focussed their attention on screening natural products to identify molecules that may serve as novel anti-osteoporotic agents.

The perennial woody plant *Eucommia ulmoides* Oliver (Eucommiaceae), which is found in China (mainly in the provinces of Yunnan, Guizhou and Sichuan) and in the United States, Japan, and some European countries (Wang et al. 2020), is valued as a rich source of polysaccharides, hemicellulose, lignin, and fatty acids and has been reported exhibit various pharmacological (e.g., antibacterial, antioxidant, immune-enhancing and anti-inflammatory) effects (Yan et al. 2018). Extracts of the cortices or leaves of *E. ulmoides* can regulate OP caused by ovariectomy (Zhang et al. 2009; 2012). Aucubin (AU) (structure in Figure S1), an iridoid glycoside present in all parts of *E. ulmoides*, and also found in *Rehmannia glutinosa* Gaertner (Orobanchaceae) and plantain [*Musa paradisiacae* Linn (Plantaginaceae)], has anti-inflammatory, antioxidant, and hepatoprotective effects (Shen et al. 2019). Our research group has demonstrated that AU slows the development of OP by promoting osteoblast differentiation in MG63 cells and in mice with dexamethasone (Dex)-induced OP (Li et al. 2018, 2020). However, the effects of AU on the osteoclast differentiation have not been systematically explored in cells or in OP mice.

In the current study, the anti-osteoporotic properties of AU related to its inhibition of osteoclast differentiation were investigated in mice in which OP had been induced by Dex injection and in RAW264.7 cells that were induced to differentiate into osteoclasts by treatment with receptor activator of nuclear factor-κB ligand (RANKL). The results showed that AU treatment of OP mice regulated their serum concentrations of osteoclast-related factors and oxidative stress-related cytokines and improved their physiological and skeletal status. AU treatment of RANKL-treated RAW264.7 cells confirmed the biochemical basis of these anti-osteoporotic effects.

## Materials and methods

### Cell culture

RAW264.7 cells (TIB-71), an immortalised murine macrophage cell line (passages <10; obtained from the American Type Culture Collection, USA), were cultured in Dulbecco’s modified Eagle’s medium (DMEM) containing 10% foetal bovine serum (FBS), 100 U/mL of penicillin, and 100 µg/mL of streptomycin, under an atmosphere of 5% CO_2_ and 95% air at 37 °C in a humidified incubator. All reagents were purchased from Gibco BRL (Grand Island, NY, USA).

### Detection of RAW264.7 cell differentiation

RAW264.7 cells were seeded into a 24-well plate at a density of 1 × 10^4^ cells/well. After 24 h incubation, cells were exposed for 7 days to normal basic media (control cells), 50 ng/mL RANKL (315-11-10) (PeproTech, USA) alone (differentiated cells), 5 µM of AU (CAS: 479-98-1, HPLC ≥ 98%) (Shanghai Yuanye Biological Technology Co., Ltd., Shanghai, China) alone, or 50 ng/mL RANKL combined with 1, 2.5, or 5 µM AU. Subsequently, the cells were stained using a Tartrate-Resistant Acid Phosphatase (TRAP) Staining Kit (44212) (Shanghai BestBio Biological Technology Co., Ltd., Shanghai, China) according to the manufacturer’s instructions. TRAP-positive cells were visualised by microscopy (Olympus, Tokyo, Japan), and those with more than three nuclei were counted as mature osteoclasts. Image-Pro Plus software (Media Cybernetics, Bethesda, Maryland, USA) was used to determine the number and area of osteoclasts.

### Western blot

RAW264.7 cells were subjected to the same treatment as above and were then lysed with radio-immunoprecipitation assay lysis buffer (Sigma-Aldrich, St. Louis, MO, USA) containing a 1% protease inhibitor cocktail (Sigma-Aldrich, St. Louis, MO, USA) and 2% phenylmethanesulfonyl fluoride (PMSF) (Sigma-Aldrich, St. Louis, MO, USA). The protein concentration of lysed cells was determined using a BCA Protein Assay Kit (Merck Millipore, Billerica, MA). The proteins were separated by 10%-12% sodium dodecyl sulphate polyacrylamide gel electrophoresis, and gels were then transferred to nitrocellulose membranes (0.45 μm) (Bio Basic, Inc., Canada) and blocked in 5% defatted milk at 4 °C for 4 h. Next, the membranes were exposed to primary antibodies at 4 °C for 12 h and then incubated with horseradish peroxidase-labeled secondary antibodies at 4 °C for 4 h. The primary antibodies used were tartrate-resistant acid phosphatase 5 (TRAP5) (bs-16578R), cathepsin K (CTSK) (bs-1611R), osteoprotegerin (OPG) (bs-20624R), haem oxygenase 2 (HO-2) (bs-1238R), superoxide dismutase 1 (SOD-1) (bs-10216R) (Bioss Inc., Beijing, China), collagen I (COL I) (ab34710), osteocalcin (OCN) (ab93876), nuclear factor erythroid 2-related factor 2 (Nrf2) (ab89443), catalase (CAT) (ab16731), SOD-2 (ab13533) (Abcam, Cambridge, MA, USA), nuclear factor of activated T-cells cytoplasmic 1 (NFATc1) (sc-7294) and β-actin (sc-47778) (Santa Cruz Biotechnology Inc., CA, USA). The secondary antibodies used were goat anti-rabbit (IH-0011) and goat anti-mouse secondary antibody (IH-0031) (Beijing Dingguo Biotechnology Co., Ltd., Beijing, China). Bands were visualised using an imaging system (BioSpectrum 600, UVP, USA), and the intensity of bands was calculated using ImageJ software (National Institutes of Health, Bethesda, MD, USA).

### Animal experimental design

The animal experiments were approved by the Animal Ethics Committee of Jilin University (SY201905006). Ninety male C57BL/6 mice (6-8 weeks old, 18-22 g in body weight; Yis Laboratory Animal Technology Co., Ltd., Changchun, China) were kept in a standard animal house at 23 ± 1 °C with a 12 h photoperiod. Food and tap water were provided *ad libitum*.

The 90 mice were randomly divided into six equal groups (*n* = 15), and all groups were subjected to experimental conditions (including agent-treatment conditions) identical to those used in our previous research (Li et al. 2020). OP was induced in mice by alternate-day intraperitoneal injections of 30 mg/kg of Dex. The positive-control mice, AU-treated mice and AU-alone-treated mice were intraperitoneally treated with 15 μg/kg of oestradiol (E2), intragastrically treated with 5 or 45 mg/kg of AU, and intragastrically treated with 45 mg/kg of AU, respectively, every other on alternate days. The entire treatment period lasted for 7 weeks, and the body weights of all mice were recorded weekly. On the last day of treatment, the mice were euthanized after the collection of peripheral blood from the caudal vein, and their femurs and internal organs (liver, spleen, kidneys and thymus) were immediately collected. The organ indexes were calculated as follows:

organ index (%) = organ weight (g)/body weight (g).

### Cytokine detection

The serum concentrations of bone gla protein (BGP) (CK-E20433M), bone morphogenetic proteins 2 (BMP-2) (CK-E20105M), bone morphogenetic protein receptor type 2 (BMPR-2) (CK-E95876M), COL I (CK-E20528M), TRAP5b (CK-E20387M), SOD-1 (CK-E20348), CAT (CK-E92636M), interleukin (IL)-1 (CK-E30418M), IL-6 (CK-E20012M), ROS (CK-E91516M) (Shanghai Yuanye Biological Technology Co., Ltd., China) and *N*-terminal propeptide of type I procollagen (P1NP) (KT30285-A) (Jiangsu Kete Biological Technology Co., Ltd., China) were determined using the corresponding enzyme-linked immunosorbent assay (ELISA) kits.

### Histological examination of organs and femur tissues

Haematoxylin and eosin (H&E) staining of femur and organ (liver, spleen and kidney) tissue, and Giemsa staining of femur tissue, were performed as described in our previous research (Li et al. 2020). A light microscope digital camera (Nikon Instruments, Tokyo, Japan) was used for histological examinations.

### Micro-computed tomography (micro-CT) analysis

The structural parameters of the trabecular and cortical regions of the femur, namely the bone surface area/bone volume (BS/BV), the bone volume/tissue volume (BV/TV), the trabecular bone mineral density (BMD), the trabecular number (Tb.N), the trabecular spacing (Tb.Sp) and the trabecular thickness (Tb.Th) were evaluated using micro-CT (µCT50, Scanco, Switzerland), and calculated by standard three-dimensional (3 D) microstructural analysis.

### Statistical analysis

All of the data are presented as the means ± standard errors of the mean (SEMs). Significant differences between the groups were determined by a one-way analysis of variance (ANOVA) followed by Tukey’s test, using SPSS 16.0 software (IBM Corporation, Armonk, NY, USA). *p* < 0.05 were considered to indicate statistical significance.

## Results

### AU relieved OP in mice

**Figure 1.**
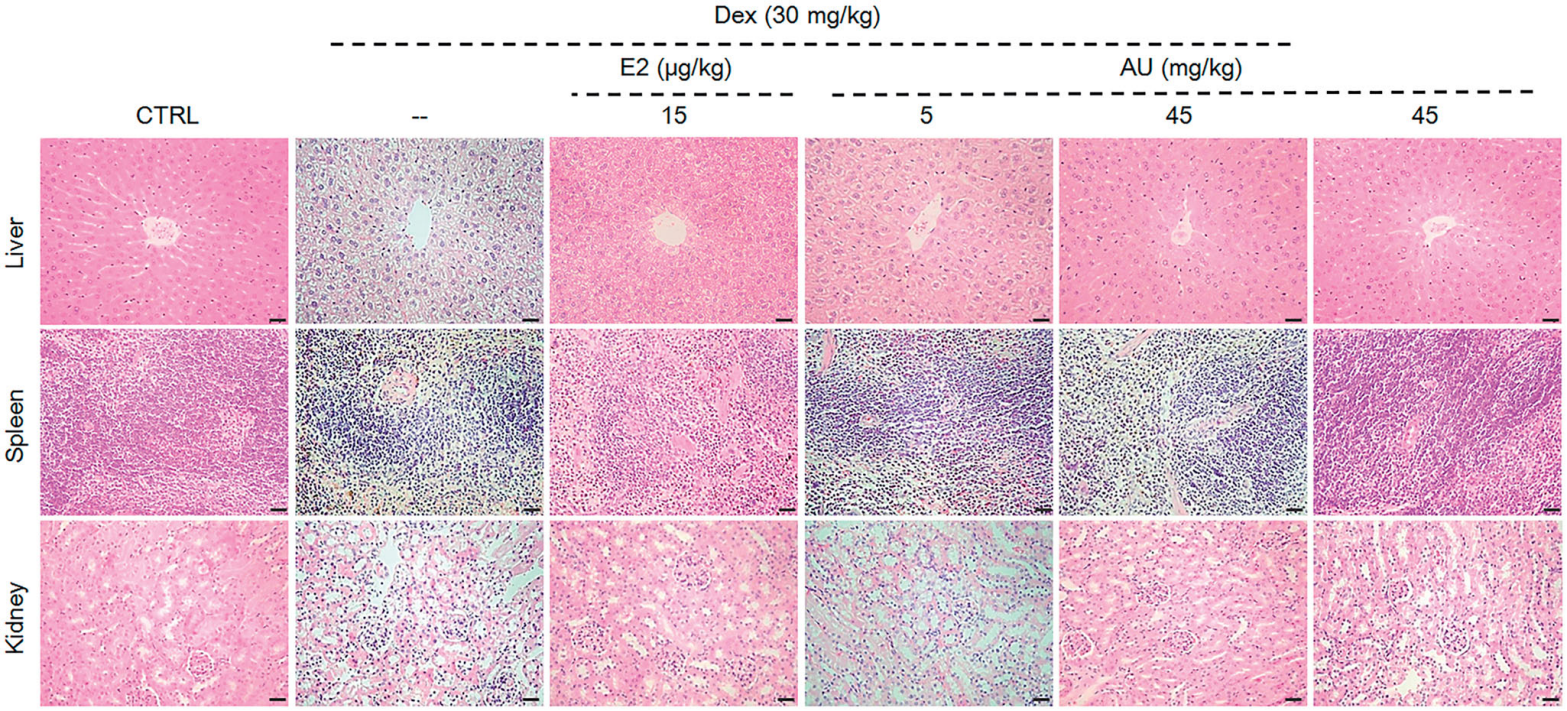
Pathological examination of haematoxylin and eosin-stained organs (liver, spleen and kidney) from osteoporosis mice (200×) (Scale Bar: 50 μm) (*n* = 3). CTRL: control; Dex: dexamethasone; E2: oestradiol; AU: aucubin.

AU failed to reverse the loss of body weight caused by Dex injection (Table 1). OP mice exhibited obvious increases in their liver index (57.1%) and decreases in their spleen index (27.8%) and thymus index (73.5%) (*p* < 0.01) (Table 1). AU treatment reversed these Dex-mediated effects on the liver and spleen index (*p* < 0.05) (Table 1) but failed to affect the thymus index (Table 1). The kidney index was not changed significantly in any of the groups (Table 1). Pathological examination revealed that interstitial edoema was present in the liver and kidneys of OP mice, and that this edoema was significantly relieved by AU treatment (Figure 1). Compared with the control mice, there were fewer areas of white pulps in the spleen of OP mice, and this decrease reversed after 7 weeks of AU treatment (Figure 1).

**Table 1.** Effects of AU on the body weight and organ indexes of OP mice.

Days	CTRL	Dex(30mg/kg)				AU (45mg/kg)
		--	E2 (15μg/kg)	AU (mg/kg)		
				5	45	
Body weight (g)						
0 day	22.4 ± 0.8	22.7 ± 0.8	22.5 ± 0.7	22.5 ± 1.0	22.3 ± 1.2	22.6 ± 0.8
7^th^ day	22.8 ± 0.9	22.4 ± 0.8	22.0 ± 0.8	22.2 ± 0.9	21.6 ± 1.0	22.8 ± 0.6
14^th^ day	23.5 ± 1.0	22.2 ± 0.9	21.7 ± 0.8	21.7 ± 1.0	21.6 ± 1.3	23.7 ± 0.9
21^st^ day	23.8 ± 1.2	22.3 ± 1.1	21.8 ± 0.9	21.9 ± 1.0	21.4 ± 1.4	24.1 ± 0.8
28^th^ day	24.5 ± 1.1	22.4 ± 1.3#	22.0 ± 1.0	22.2 ± 1.2	22.0 ± 1.4	25.1 ± 1.1
35^th^ day	25.1 ± 1.3	21.6 ± 1.8#	20.9 ± 1.1	22.0 ± 1.7	21.3 ± 1.5	26.0 ± 1.3
42^nd^ day	25.8 ± 1.3	21.8 ± 1.5##	21.6 ± 0.8	21.6 ± 1.5	21.7 ± 1.5	26.4 ± 1.2
49^th^ day	26.7 ± 1.4	22.2 ± 1.1##	21.9 ± 0.8	21.6 ± 0.9	21.0 ± 1.7	26.7 ± 1.1
Organ Index (%)						
Liver	4.96 ± 0.43	7.79 ± 0.24##	7.51 ± 0.26	7.70 ± 0.64	6.9 ± 0.77*	4.38 ± 0.78
Spleen	0.36 ± 0.04	0.26 ± 0.01##	0.26 ± 0.08	0.32 ± 0.09**	0.27 ± 0.06	0.28 ± 0.07
Kidney	1.63 ± 0.20	1.55 ± 0.05	1.57 ± 0.08	1.60 ± 0.09	1.39 ± 0.45	1.38 ± 0.20
Thymus	0.223 ± 0.07	0.059 ± 0.03##	0.073 ± 0.02*	0.06 ± 0.03	0.05 ± 0.04	0.32 ± 0.52

The data were analysed using a one-way ANOVA and expressed as means ± SEM (*n* = 15). #*p* < 0.05 and ##*p* < 0.01 versus control mice; *p < 0.05 and **p < 0.01 versus OP mice. CTRL: control; Dex: dexamethasone; E2: oestradiol; AU: aucubin.

**Figure 2.**
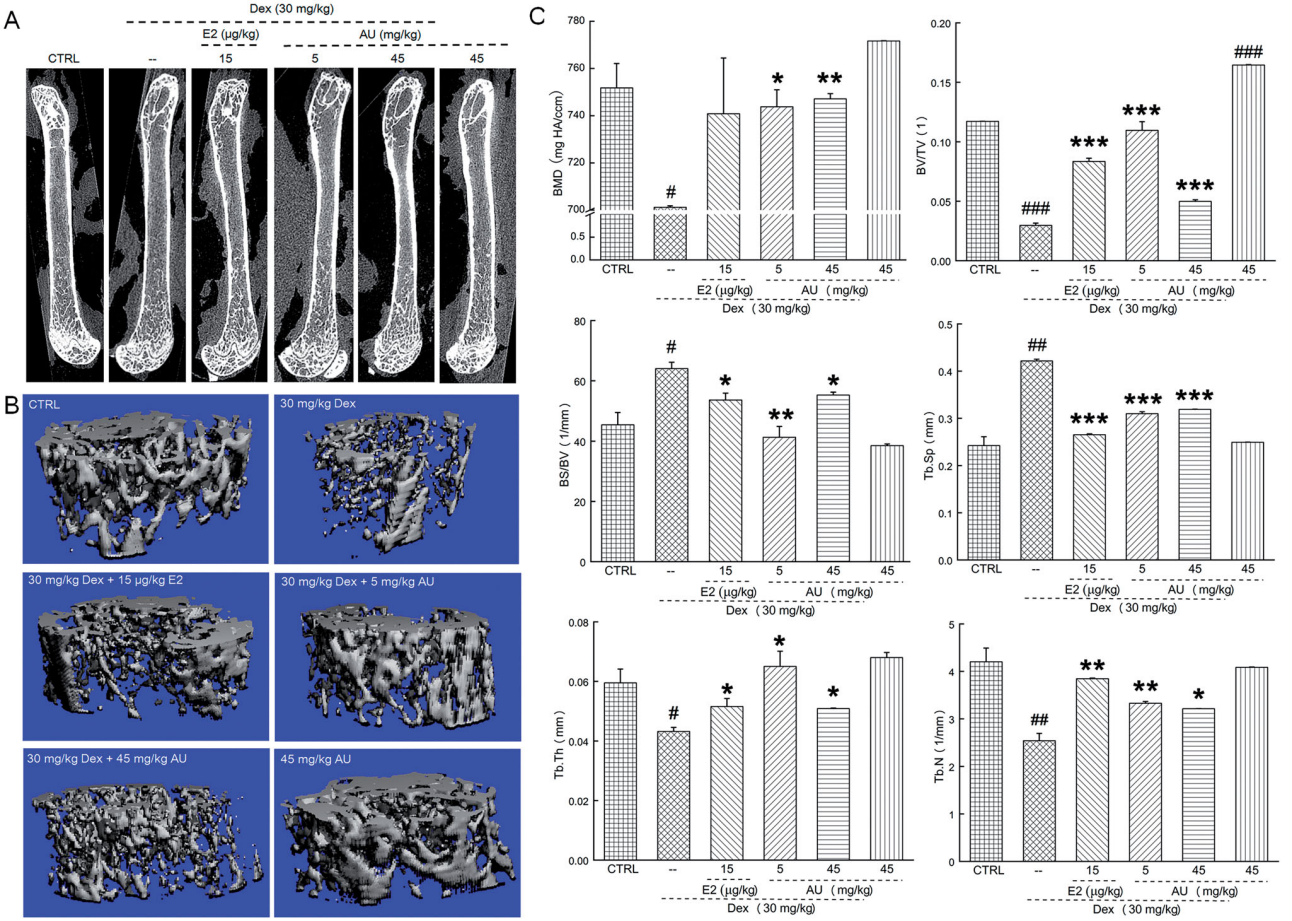
Effects of AU on the femoral bone morphology and the concentrations of osteoporosis (OP)-related factors in OP mice. (A) Micro-computed tomography images of the femurs of OP mice. (B) Three-dimensionally reconstructed images of trabecular bone in the femurs of OP mice. (C) Analysis of OP parameters (BMD, BV/TV, BS/BV, Tb.Sp, Tb.Th and Tb.N). Data are expressed as the means ± SEMs (*n* = 6) and were analysed using a one-way ANOVA. #*p* < 0.05, ##*p* < 0.01 and ###*p* < 0.001 versus control mice; *p < 0.05, **p < 0.01 and ***p < 0.001 versus OP mice. CTRL: control; Dex: dexamethasone; E2: oestradiol; AU: aucubin.

The most prominent feature of OP that is caused by the imbalance between bone formation and bone resorption is the degradation of bone quality and the decrease of bone density (Vijayan et al. 2014). As cortical thickness correlates with bone strength and the probability of bone fracture (Ohlsson et al. 2018), micro-CT was used to analyse the parameters of bone development in the femur. In the femurs of OP mice, compared to those of control mice, the cortical bone layer was thinned and the trabecular bone density was decreased (Figure 2(A,B)). However, after 7 weeks of AU treatment, the thickness of cortical bone and the number of trabeculae were increased in the femurs of OP mice (Figure 2(A,B)). In addition, 3 D imaging analyses of the femurs of OP mice indicated that AU treatment increased the BMD (>6.08%) (*p* < 0.05) [BMD refers to the amount of mineral density in bone tissue and can be used to assess the risk of fracture (Kanis et al. 2008)], the BV/TV (>68.6%) (*p* < 0.001), the Tb.Th (>17.8%) (*p* < 0.05) and the Tb.N (>28.0%) (*p* < 0.05) [Tb.N and Tb.Th reflect the number and thickness of trabeculae (Levy et al. 2015)] and decreased the BS/BV (>13.6%) (*p* < 0.05) [BV/TV and BS/BV reflects changes in bone mass and can be used to evaluate the strength of bone trabeculae (Georgiou et al. 2018)] and the Tb.Sp (>35.5%) (*p* < 0.001) (Figure 2(C)) [Tb.Sp reflects the separation of trabeculae and they reflect the spatial structure of trabeculae together (Takahashi et al. 2016)].

**Figure 3.**
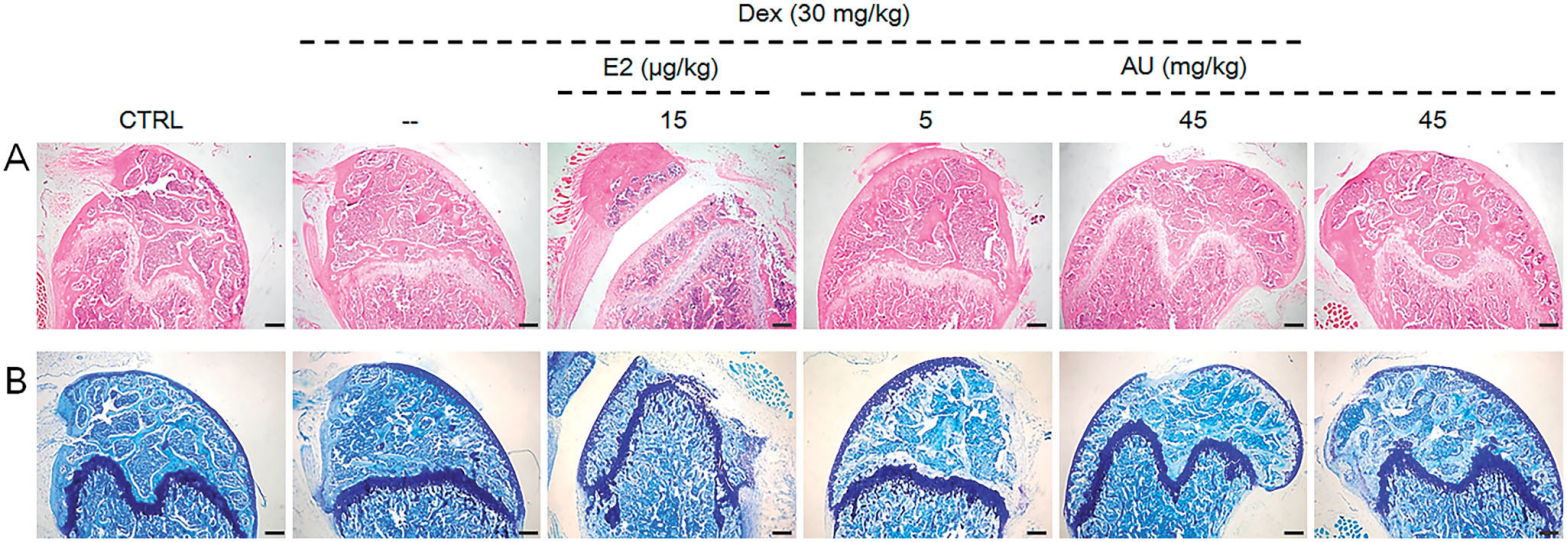
The effects of AU on the femoral histology of OP mice, as detected by (A) haematoxylin and eosin staining (40×) (Scale Bar: 200 μm) and (B) Giemsa staining (40×) (Scale Bar: 200 μm) (*n* = 6). CTRL: control; Dex: dexamethasone; E2: oestradiol; AU: aucubin.

In the femurs of AU-treated OP mice, compared to those of non-AU-treated OP mice, the number of trabeculae was increased, which increased the strength of the bone (Figure 3(A)), and the loss of chondrocytes was relieved (Figure 3(B)).

**Figure 4.**
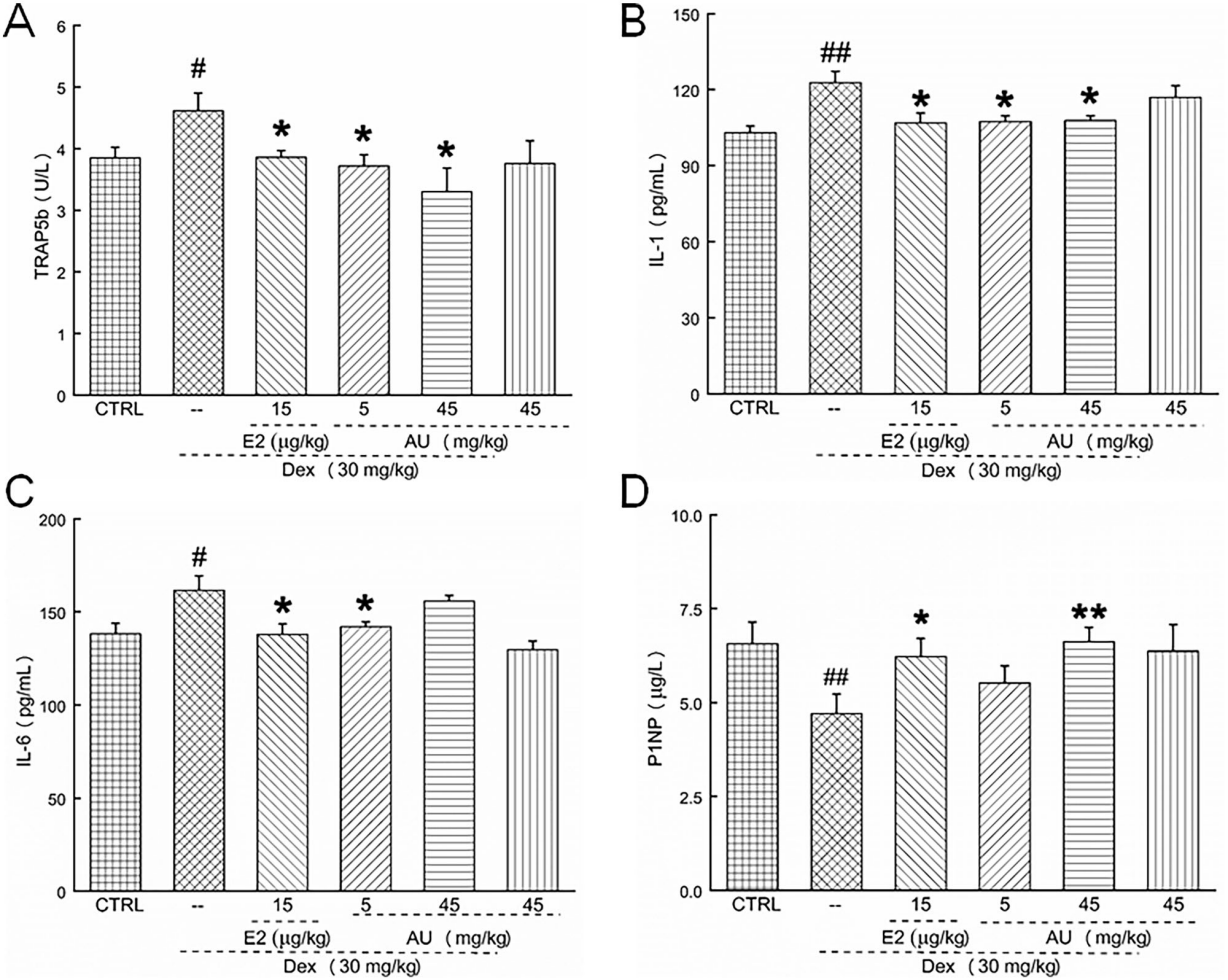
AU regulated the serum concentrations of (A) TRAP5b, (B) IL-1, (C) IL-6 and (D) P1NP in osteoporosis mice. Data are expressed as the means ± SEMs (*n* = 6) and were analysed using a one-way ANOVA. #*p* < 0.05 and ##*p* < 0.01 versus control mice; *p < 0.05 and **p < 0.01 versus OP mice. CTRL: control; Dex: dexamethasone; E2: oestradiol; AU: aucubin.

**Figure 5.**
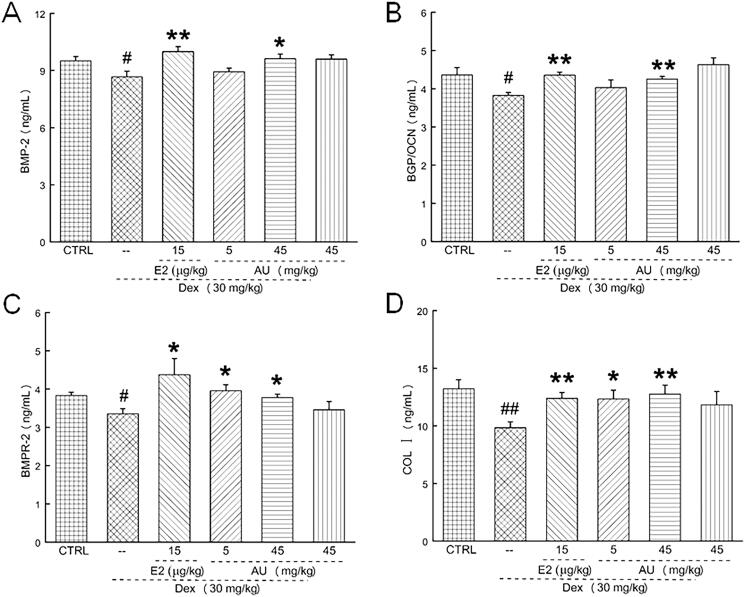
AU increased the concentration of osteogenic differentiation-related factors in the serum of osteoporosis mice. Thus, AU enhanced the serum concentrations of (A) BMP-2, (B) BGP, (C) BMPR-2 and (D) COL I. Data are expressed as the means ± SEMs (*n* = 6) and were analysed using a one-way ANOVA. #*p* < 0.05 and ##*p* < 0.01 versus control mice; *p < 0.05 and **p < 0.01 versus OP mice. CTRL: control; Dex: dexamethasone; E2: oestradiol; AU: aucubin.

In OP mice, AU markedly decreased the serum concentrations of TRAP5b (a characteristic marker of osteoclast activity that is present in the ruffled border of osteoclasts) (>19.6%) (*p* < 0.05) (Figure 4(A)), IL-1 [an increase in the serum concentrations of pro-inflammatory cytokines, e.g., autocrine cytokines such as IL-1, accelerates bone loss (Luo et al. 2014)] (>12.2%) (*p* < 0.05) (Figure 4(B)) and IL-6 [a pro-inflammatory cytokine related to bone metabolism (Bogusław et al. 2010)] (12.1%) (*p* < 0.05) (Figure 4(C)), and increased the serum concentration of P1NP (a bone formation marker [25]) (40.4%) (*p* < 0.01) (Figure 4(D)). These results demonstrated that AU inhibited osteoclast differentiation and promoted osteoblast differentiation. In addition, similar to our previous results (Li et al. 2020), AU markedly increased the serum concentrations of BMP-2 (11.6%) (*p* < 0.05) (Figure 5(A)), BGP (11.3%) (*p* < 0.01) (Figure 5(B)), BMPR-2 (>12.5%) (*p* < 0.05) (Figure 5(C)) and COL I (>25.5%) (*p* < 0.05) (Figure 5(D)), which confirmed its promotion of osteoblast differentiation.

**Figure 6.**
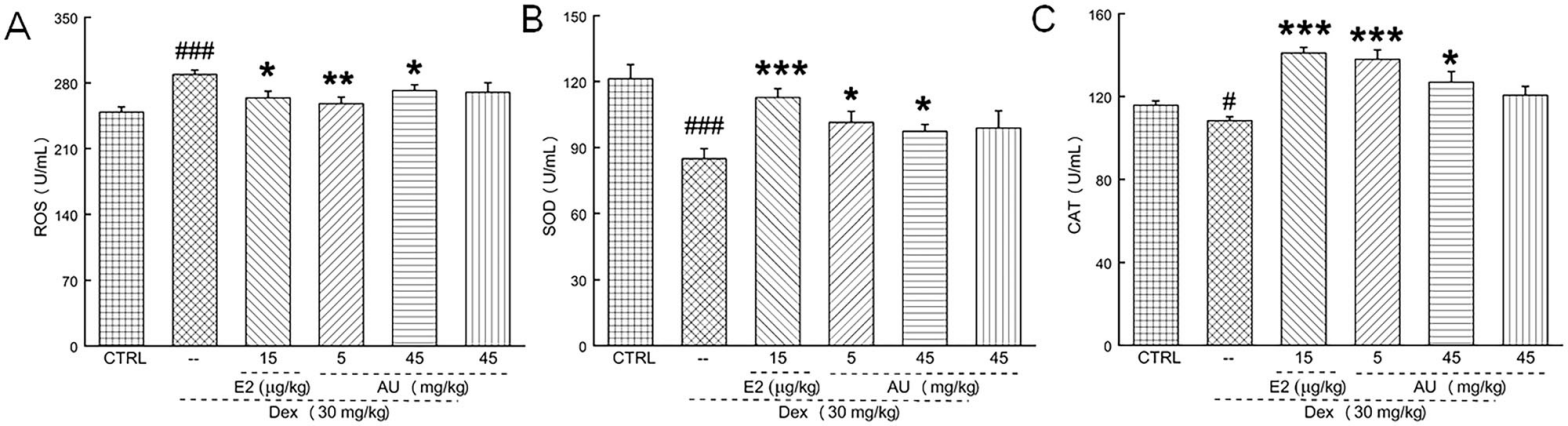
AU regulated the concentrations of oxidative stress-related factors in the serum of OP mice. AU decreased the concentrations of (A) ROS and increased the concentrations of (B) SOD and (C) CAT. Data are expressed as the means ± SEMs (*n* = 6) and were analysed using a one-way ANOVA. #*p* < 0.05 and ###*p* < 0.001 versus control mice; *p < 0.05, **p < 0.01 and ***p < 0.001 versus OP mice. CTRL: control; Dex: dexamethasone; E2: oestradiol; AU: aucubin.

ROS produced by oxidative stress can stimulate osteoclast differentiation (Zhu et al. 2018). It was found that 7-week AU treatment of OP mice significantly decreased their serum concentrations of ROS (>5.9%) (*p* < 0.05) (Figure 6(A)), and increased their serum concentrations of SOD (>14.6%) (*p* < 0.05) (Figure 6(B)) and CAT (>17.2%) (*p* < 0.05) (Figure 6(C)).

Compared with control mice, no significant changes on body weight or organ indexes (Table 1), organ structure (Figure 1), bone morphology or structure (Figures 2 and 3), or concentration of serum cytokines (Figures 4, 5 and 6) were observed in AU only-treated healthy mice. These results also illustrated that the median lethal dose (LD_50_) of AU in these mice was much greater than 45 mg/kg.

### AU suppressed RANKL-induced osteoclast differentiation of RAW264.7 cells via Nrf2 signalling

**Figure 7.**
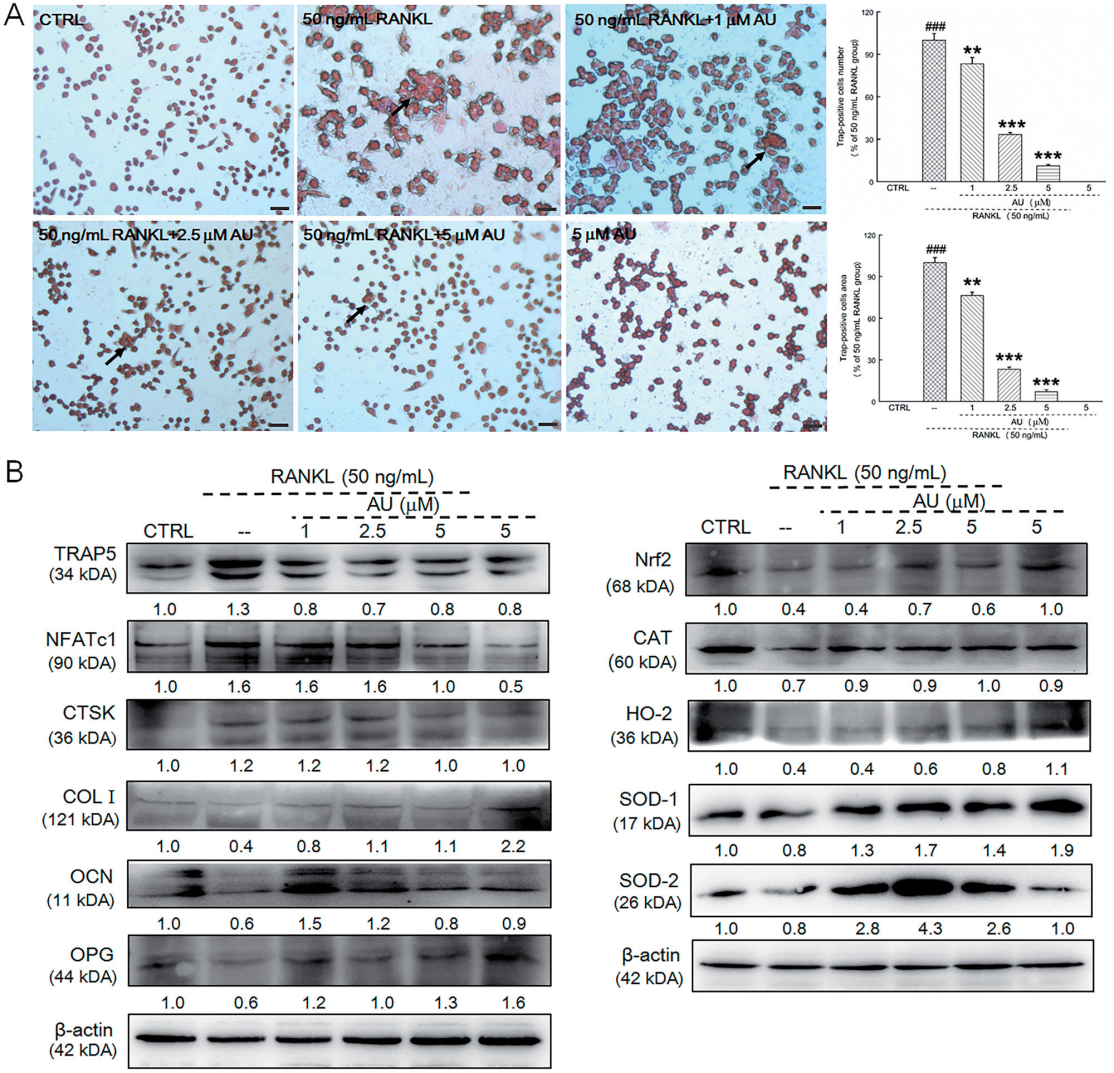
AU suppressed the receptor activator of nuclear factor-κB ligand (RANKL)-induced differentiation of RAW264.7 cells to osteoclasts. (A) AU decreased the percentage of numbers and area of TRAP-positive RANKL-exposed RAW264.7 cells (200×) (Scale Bar: 50 μm). Data are expressed as the means ± SEMs (*n* = 6) and were analysed using a one-way ANOVA. ###*p* < 0.001 versus control cells; **p < 0.01 and ***p < 0.001 versus RANKL-induced differentiated cells. (B) In RANKL-exposed RAW264.7 cells, AU increased the expression levels of COL I, OCN, OPG, Nrf2, CAT, HO-2, SOD-1 and SOD-2, and decreased the expression levels of TRAP5, NFATc1 and CTSK. The quantitative expression of each protein was normalised to β-actin and is presented as the fold-expression compared with the expression in control cells (*n* = 6). CTRL: control; AU: aucubin.

RAW264.7 cells, which belong to the mouse macrophage cell line, can be induced to differentiate into osteoclasts under various conditions (Chen et al. 2015). RANKL is a key factor for osteoclast formation (Nakashima et al. 2011), and it was found that the treatment of RAW264.7 cells with RANKL led to an increase in the number and area of multinuclear TRAP-positive cells (*p* < 0.001), indicating that the RAW264.7 cells had differentiated into osteoclasts (Figure 7(A)). When RAW264.7 cells were co-treated with AU and RANKL, compared with the TRAP-positive cells in the RANKL treatment group, the proportion of TRAP-positive cells decreased from 83.3% to 11.1% (*p* < 0.01), and the area proportion of TRAP-positive cells decreased from 76.3% to 7.1% (*p* < 0.01), indicating that AU treatment had caused a decrease in the number and area of multinucleated osteoclasts (Figure 7(A)). In contrast, treatment of RAW264.7 cells with AU alone failed to influence their morphology (Figure 7(A)).

Oxidative stress can lead to bone loss, resulting in an imbalance between osteogenesis and osteolysis, and the development of OP (Ma et al. 2012). Compared with untreated RAW264.7 cells, RANKL treatment increased the expression levels of TRAP5 (30.0%), NFATc1 (60.0%) and CTSK (20.0%) and decreased the expression levels of COL I (60.0%), OCN (40.0%), and OPG (40.0%) (Figure 7(B)). Furthermore, RANKL treatment decreased the expression levels of Nrf2 (60.0%) and its downstream proteins, namely CAT (30.0%), HO-2 (60.0%), SOD-1 (20.0%) and SOD-2 (20.0%) (Figure 7(B)). However, RAW264.7 cells that were co-treated with AU exhibited none of these altered expression levels (Figure 7(B)).

## Discussion

In a recent study, we confirmed that AU could slow the development of OP in mice by promoting osteogenesis (Li et al. 2020). In the current study, we focussed on examining the inhibition of osteoclast differentiation by AU.

Glucocorticoids are a class of steroid hormones secreted by the adrenal cortex that can accelerate bone flow patterns. In particular, glucocorticoids can cause damage to the trabecular bone, which in severe cases can result in OP (Compston 2018). Glucocorticoids can also increase osteoclast proliferation by increasing the activity of macrophage colony-stimulating factor (M-CSF) and RANKL (Swanson et al. 2006). This study showed that 7-week treatment with AU markedly improved the femoral structure of OP mice (in which OP had been established by long-term injection of Dex), and this was confirmed by micro-CT and 3 D imaging analyses.

Bone homeostasis is a dynamic process controlled by a balance between osteoblasts and osteoclasts, which results in bone being continuously consumed and regenerated, and an adequate concentration of calcium ions being maintained in the blood (Chen et al. 2017). The diagnosis of bone marker concentrations is essential to evaluate the development of OP. As P1NP is a marker of bone formation, it can be used as a high-sensitivity predictor of the state of bone metabolism. Specifically, during the formation of bone fibres, equal concentrations of P1NP and COL I are released into the blood, and thus, P1NP concentration can reflect the level of osteogenesis (Kanis et al. 2008; Ohishi et al. 2017). In this current study, 7 weeks AU treatment significantly increased the serum concentrations of P1NP in OP mice relative to control mice, which preliminarily proves that AU slowed the development of OP by increasing bone formation.

TRAP is specifically distributed in the cytoplasm of osteoclasts (Liu et al. 2019), and during bone resorption, TRAP participates in the degradation of solid calcium phosphate substrates in the bone matrix. Type 5 TRAP has two subtypes, TRAP5a and TRAP5b (Ma et al. 2012). TRAP5b is mainly derived from osteoclasts and is thus a marker of bone resorption that can reflect the number of osteoclasts and the status of bone metabolism (Nishikawa et al. 2016). The activity of TRAP5 is positively related to the number of osteoclasts, and thus, this number can be regarded as a bone resorption parameter that can be used to analyse the state of osteoclasts in the body (Motyl and McCabe 2009). In this current study, AU markedly decreased the concentrations of TRAP5 in OP mice and RANKL-treated RAW264.7 cells, indicating that AU inhibited osteoclasts differentiation in these systems.

IL-1 is a pro-inflammatory cytokine that can regulate the differentiation of osteoclasts by stimulating the expression of IL-6, which enhances the activity of osteoclasts and promotes the expression of RANKL (Al-Daghri et al. 2017). RANKL then binds to the receptor activator of nuclear factor-κB (RANK) receptor on the osteoclast precursor cell membrane, which activates specific signal transduction pathways to regulate the expression of osteoclast genes and initiate osteoclast differentiation (Zhong et al. 2019). As a transcription factor that regulates osteoclast differentiation, NFATc1 immediately stimulates the expression of osteoclast-related genes, including TRAP and CTSK (Li et al. 2019). CTSK is a protease that can lyse parts of the bone matrix proteins, thereby affecting bone metabolism and promoting bone loss (Wu et al. 2018). Consistent with the other results of this current study, AU significantly reduced the serum concentrations of IL-1 in OP mice, what’s more, AU decreased the expression of NFATc1, TRAP5 and CTSK in RANKL-exposed RAW264.7 cells.

OPG, which is also called osteoclastogenesis inhibitory factor, acts together with RANKL to inhibit osteoclast differentiation and bone resorption (Xu et al. 2018; Mo et al. 2019). COL I stimulates the transcription of osteoblast-formation genes and is the main component of the skeletal matrix, and CTSK degrades COL I (Qiu et al. 2015; Drake et al. 2017). In addition, a combination of BMP and its receptor (BMPR) regulate bone formation (Hu et al. 2018), and BGP maintains a normal rate of bone calcification and inhibits the rate of cartilage mineralisation, which is an indicator of osteoblast activity (Zhou et al. 2019). In the current study, AU treatment increased the serum concentrations of BMP-2, BGP, BMPR-2 and COL I in OP mice and increased the expression levels of OPG, OCN and COL I in RANKL-exposed RAW264.7 cells. Thus, AU increased osteoblast differentiation and decreased osteoclast differentiation.

Oxidative stress results from an imbalance of oxidation and antioxidation, and as this promotes osteoclast differentiation, oxidative stress is responsible for the development of OP (Manolagas 2010). Similarly, inflammatory and metabolic disorders lead to excessive generation of ROS, which increases the pathological state of the body (Bonaccorsi et al. 2018). ROS also activate the transcription factor forkhead box O (FoxO), which attenuates osteoblast formation and aggravates bone cell apoptosis (Almeida et al. 2017). Nrf2 is a core transcription factor that resists oxidative stress and regulates the activation and expression of downstream antioxidant elements to maintain cell homeostasis (Zhang et al. 2015). A deficiency in Nrf2 increases the concentration of RANKL in serum and promotes osteoclast proliferation, which affects the mineralisation of osteoblasts (Ibáñez et al. 2014). In normal physiological conditions, Nrf2 and Keap1 form a dimer in the cytoplasm, which inhibits the function of Nrf2. In a state of peroxidation, this Nrf2–Keap1 dimer dissociates, and Nrf2 then binds to musculoaponeurotic fibrosarcoma (Maf) proteins to activate the expression of antioxidant genes that are dependent on antioxidant response elements (Tu et al. 2019). SOD converts the superoxide radical anion to H_2_O_2_, which is further decomposed by CAT. SOD-1 is present in the cytoplasm, whereas SOD-2 is present in the mitochondria (Zhang et al. 2015). The abnormal expression of ROS promotes the expression of HO-2, which protects against damage due to oxidative stress (Chen et al. 2019). Oestrogen also affects HO-2 expression (Chen et al. 2019). Thus, in this current study, the antioxidative effects of AU in OP mice and RANKL-exposed RAW264.7 cells may be due to AU regulation of Nrf2 and its downstream proteins.

It is noteworthy that AU slowed the development of OP in a non-dose dependent manner. This is primarily because AU not only promotes the formation of osteoblasts, but also inhibits the development of osteoclasts. However, our data did not reveal which function of AU was most responsible for its anti-osteoporotic effects, and this will be investigated in future research.

## Conclusion

This study showed that the treatment of OP mice with AU slowed the development of OP by inhibiting the differentiation of osteoclasts. Furthermore, AU treatment of RAW264.7 cells that had been induced to differentiate into osteoclasts by RANKL treatment showed that AU achieved its anti-osteoporotic effects (at least partly) by triggering Nrf2-mediated antioxidation.

## Supplementary Material

Supplemental Material

## Data Availability

All data generated and analysed during the study are included in this published article.
