# Supplementary figures and images for: Aucubin slows the development of osteoporosis by inhibiting osteoclast differentiation via the nuclear factor erythroid 2-related factor 2-mediated antioxidation pathway
Source: Pharm Biol. 2021 Nov 10;59(1):1554–63. doi: 10.1080/13880209.2021.1996614 (PMC8583775; doi:10.1080/13880209.2021.1996614)

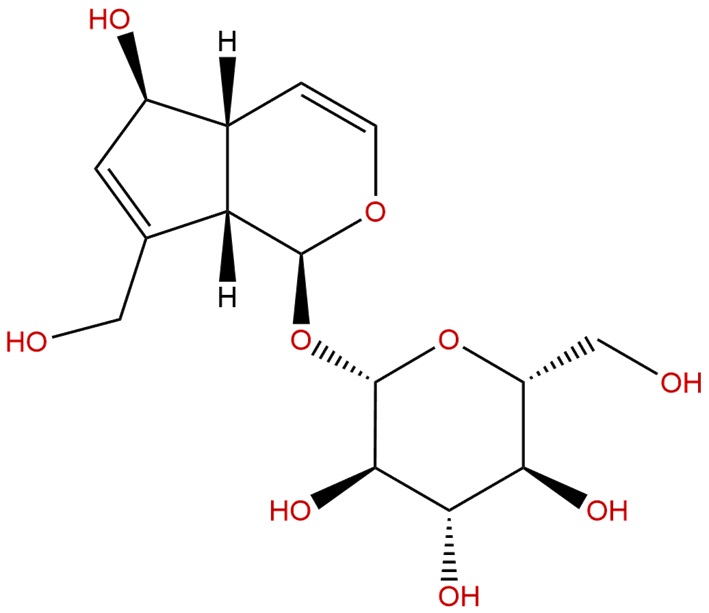

Supplement: Supplemental Material [file IPHB_A_1996614_SM5181.jpg]
